# Glacial Ice Age Shapes Microbiome Composition in a Receding Southern European Glacier

**DOI:** 10.3389/fmicb.2021.714537

**Published:** 2021-11-11

**Authors:** Eva Garcia-Lopez, Ana Moreno, Miguel Bartolomé, Maria Leunda, Carlos Sancho, Cristina Cid

**Affiliations:** ^1^Molecular Evolution Department, Centro de Astrobiologia (CSIC-INTA), Madrid, Spain; ^2^Departamento de Procesos Geoambientales y Cambio Global, Instituto Pirenaico de Ecología—CSIC, Zaragoza, Spain; ^3^Departamento de Geología, Museo de Ciencias Naturales—CSIC, Madrid, Spain; ^4^Oeschger Centre for Climate Change Research, Institute of Plant Sciences, University of Bern, Bern, Switzerland; ^5^Swiss Federal Research Institute for Forest, Snow and Landscape Research WSL, Birmensdorf, Switzerland; ^6^Departamento de Ciencias de la Tierra, Universidad de Zaragoza, Zaragoza, Spain

**Keywords:** mountain glacier, microbial community profiling, next-generation sequencing, environmental variables, Pyrenees, statistical approaches, temporal dynamics

## Abstract

Glaciers and their microbiomes are exceptional witnesses of the environmental conditions from remote times. Climate change is threatening mountain glaciers, and especially those found in southern Europe, such as the Monte Perdido Glacier (northern Spain, Central Pyrenees). This study focuses on the reconstruction of the history of microbial communities over time. The microorganisms that inhabit the Monte Perdido Glacier were identified using high-throughput sequencing, and the microbial communities were compared along an altitudinal transect covering most of the preserved ice sequence in the glacier. The results showed that the glacial ice age gradient did shape the diversity of microbial populations, which presented large differences throughout the last 2000 years. Variations in microbial community diversity were influenced by glacial conditions over time (nutrient concentration, chemical composition, and ice age). Some groups were exclusively identified in the oldest samples as the bacterial phyla Fusobacteria and Calditrichaeota, or the eukaryotic class Rhodophyceae. Among groups only found in modern samples, the green sulfur bacteria (phylum Chlorobi) stood out, as well as the bacterial phylum Gemmatimonadetes and the eukaryotic class Tubulinea. A patent impact of human contamination was also observed on the glacier microbiome. The oldest samples, corresponding to the Roman Empire times, were influenced by the beginning of mining exploitation in the Pyrenean area, with the presence of metal-tolerant microorganisms. The most recent samples comprise 600-year-old ancient ice in which current communities are living.

## Introduction

Glaciers are considered authentic ecosystems inhabited by microorganisms that maintain active biochemical routes and play a key role in biogeochemical cycles (Boetius et al., 2015; Garcia-Lopez et al., 2016; Anesio et al., 2017). All these microorganisms, especially bacteria and algae, are the basis of food webs that allow the life of more complex organisms, such as cold-tolerant insects and copepods within glacial ice (Kohshima et al., 2002). Glaciers constitute natural traps of both organic (microorganisms, pollen, etc.) and inorganic material (atmospheric dust, suspended particles, etc.) deposited over time that makes them excellent archives of past climate and environmental changes. The identification of microorganisms in glacier perforations has profusely been carried out in glaciers in the Arctic and Antarctic regions and also at mid-latitude mountains. In the Arctic region, the diversity and survival of microorganisms trapped in the ice of several Greenland glaciers at least 120,000 years ago have been reported (Miteva et al., 2004). Other reports identified microorganisms in Greenland glacier samples between 500 and 157,000 years before present. In these cores, members of Firmicutes and Cyanobacteria were the most prevalent bacteria, and Rhodotorula species were the most common eukaryotic representatives (Knowlton et al., 2013). Bacteria preserved in an Alaskan ice wedge were also analyzed by cultivation and molecular techniques (Katayama et al., 2007). These microorganisms adapted to the frozen conditions have survived for 25,000 years. In Antarctica, several ice core sections (between 500 and 70,000 years before present) were analyzed from the Byrd glacier (Knowlton et al., 2013). Furthermore, bacterial isolates were cultured belonging to different lineages from the ice accreted below glacial ice of Lake Vostok (Christner et al., 2001). The Antarctic coastal glaciers have also been drilled in search of cultivable and physiologically active organisms (Antony et al., 2012; Martinez-Alonso et al., 2019). In these reports, the highest numbers of microorganisms in both Arctic and Antarctic ice cores were found in sections that were deposited during ancient times of low atmospheric CO_2_, low global temperatures, and low levels of atmospheric dust (Knowlton et al., 2013). Furthermore, in mountain glaciers, snow algae were identified from ice cores recovered in Nepal (Yoshimura et al., 2000), as well as bacterial isolates were collected from the Tibetan Plateau (Zhang et al., 2008; Shen et al., 2018).

**FIGURE 1:**
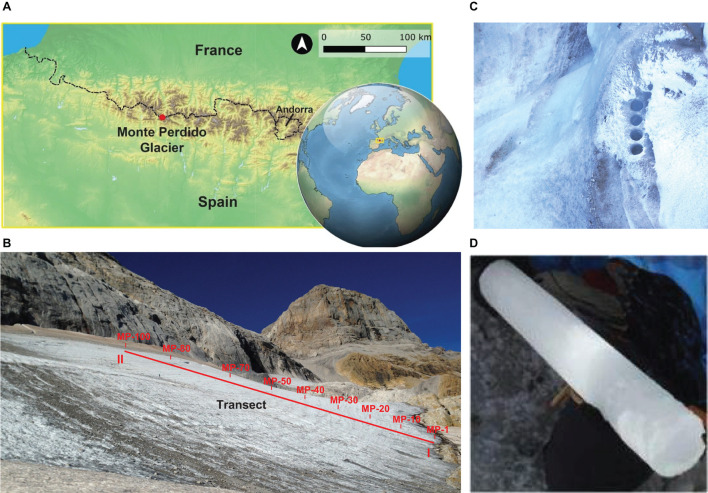
Geological setting of samples collected at Monte Perdido glacier. **(A)** Location of MPG. **(B)** Picture of Monte Perdido glacier where altitudinal transect is indicated. Sampling points go from the oldest (MP1) to the modern (MP100). **(C)** Detailed view indicating that every sample consisted of three to four drilled cylinders. **(D)** Example of an ice core.

Thus, up to now, other glaciers have been investigated, but in general, the reported results refer to cores taken at greater depth, and therefore, they were older than Monte Perdido Glacier (MPG). Furthermore, most of the reports refer to glaciers in the Polar Regions. Little is known about the microbiology of glaciers in southern Europe, such as those in the Pyrenees. Nowadays, Pyrenean glaciers are rapidly disappearing due to the increasing temperatures caused by the current climate warming and their study from all points of views is an urgent need (García-Ruiz et al., 2014; López-Moreno et al., 2016, 2019). This work focuses on investigating firstly whether there is a true correlation between the age of the ice samples and the microbial diversity of the strata populations and secondly whether Glacial ice age shapes microbiome composition and finally whether there is any type of human influence on the microbial population, since the current MPG is only 2000 years old. Glaciers have shown large fluctuations in their size in relation to changing climatic conditions during the Quaternary following the glacial–interglacial cycles. Mountain glaciers, in particular, sensitively responded to rapid climate oscillations during the Last Glacial Cycle (130,000–14,000 years) (Preusser et al., 2003; Davis et al., 2009; Lewis et al., 2009; Schoeneich, 2011; Bartolomé et al., 2021). Contrarily, the current interglacial period (the Holocene; last 11,700 years) has been considered a rather stable climatic period. Still, different climatic changes have been identified along the Holocene and, accordingly, glacier fluctuations were recorded (García-Ruiz et al., 2014; López-Moreno et al., 2016, 2019). The end of the Holocene Thermal Maximum (HTM) (6,000–5,000 years ago) led to the beginning of the Neoglacial cooling (Davis et al., 2009; Kumar, 2011), when many glaciers expanded. From that time, mountain glaciers mostly receded until the Little Ice Age (LIA), a cold period that started in the thirteenth century reaching minimum temperatures around the 19th century (Solomina et al., 2015) and that was characterized by a general glacier expansion (Oliva et al., 2018). The progressive global temperature drop identified after the HTM makes current temperatures the warmest in the last 6,500 years (Kaufman et al., 2020). Glaciers all over the world, including those from the Pyrenees, showed a vast retreat after the LIA (Garcia-Lopez and Cid, 2017). Thus, a historically unprecedented glacier regression is nowadays occurring all over the planet (Marzeion et al., 2014; Zemp et al., 2015). The Pyrenean range, located in the north east of the Iberian Peninsula (Figure 1A), hosts some of the southernmost mountain glaciers of Europe (Grunewald and Scheithauer, 2010). Glaciers from southern Europe are among the most threatened by global warming (López-Moreno et al., 2019). In the Pyrenees, since the beginning of the 20th century, the temperature has increased 1.3°C (OPCC-CTP, 2018), and therefore, the Pyrenean glaciers are experiencing a rapid retreat (García-Ruiz et al., 2014; López-Moreno et al., 2016, 2019; Rico et al., 2017; Vidaller et al., 2021). Thus, it is necessary to study them before they disappear completely. A recent study (Moreno et al., 2021) presents the chronology of MPG (∼0–1,200 years CE), which is located in the north face of the Monte Perdido peak in Ordesa y Monte Perdido National Park, Spain (42∘40′50″N; 0∘02′15″E; 3355 m a.s.l.) (Figure 1).

In previous investigations, we had already identified eukaryotic microorganisms on the surface of this glacier (Garcia-Descalzo et al., 2013). In the present paper, we deepen our knowledge of the microbial populations that inhabit the glacier ice (using samples obtained along an altitudinal transect covering most of the preserved ice sequence, Figure 1B); and we compare the distribution of these populations with the chemical characteristics of the ice samples. Additionally, we establish a comparison of the populations in relation to the age of the samples, which had previously been dated (Moreno et al., 2021).

The results obtained in this study allow interesting questions to be discussed: (i) what microbial lineages are inhabiting the glacier ice today?; (ii) is there a diversity gradient in microbial population composition across the glacier?; (iii) can certain microbial lineages be used as proxy for a chronosequence?; (iv) could a chronosequence be established, based on the set of microbial populations?; (v) are microbial populations influenced by human activities around the glacier?

## Materials and Methods

### Ice Drilling, Sampling, and Chronological Approach

Glacial ice samples were cored in September 2017 as part of the PaleoICE EXPLORA project (Moreno et al., 2021). A summary of the overall experimental strategy is represented in Supplementary Figure 1. Ice samples were collected in an ordered chrono-stratigraphical sequence covering from the oldest to the newest ice preserved in the glacier (Figure 1B). A total of 100 samples, every one constituted by three to four cores (6 cm in diameter and 25 cm in length), were recovered using a custom stainless steel crown adaptor on a cordless power drill (Figures 1C,D). For this study, only 27 of these cores representing nine samples were used (MP1, MP10, MP20, MP30, MP40, MP50, MP70, MP80, and MP100, with three replicates each, named a, b, c). These 27 replicates were individually melted and analyzed (Supplementary Table 1). Protocols for aseptic sampling and tracing of potential contaminations were applied following previously published methods (Martinez-Alonso et al., 2019). To control the contamination during the laboratory work, 500 ml of MilliQ water was analyzed using identical procedures. The meltwater samples were individually filtered through sterile filters (Millipore) with pores of 0.22 μm attached to a vacuum pump in a flow hood, previously sterilized with ethanol. The fractions obtained in the filters were used for DNA extraction and the filtered waters were used for the chemical analyses.

Ice chronology along the sampled transect was established elsewhere (Moreno et al., 2021) by a combination of different radiometric, including ^210^Pb, ^137^Cs, ^14^C, and water-insoluble organic carbon (WIOC) technique. Additionally, this absolute chronology was evaluated by the comparison with a nearby paleoenvironmental reconstruction obtained from Marboré Lake where anthropogenic metals (Zn, Se, Cd, Hg, Pb) were measured. Through this comparison, it was evident the lack of ice from the Industrial Era and, eventually, a depth-age model was established (Moreno et al., 2021).

### Chemical Analysis of Meltwater

The geochemistry of every ice core sample was characterized using several complementary techniques.

In these experiments, sample replicates different from those used for DNA extraction were used. The concentration of insoluble particles (Supplementary Table 1) was calculated by filtering individual samples of 100 ml of meltwater through tared filters with pores of 0.45 μm attached to a vacuum pump. The suspended matter caught up on the filter was measured in mg/100 ml of solution. Then, the dried filters were weighed.

Basic measurements of physical and chemical parameters of meltwater were made with a calibrated pH and salinity meter (WTW, Weilheim, Germany).

Assays for NH_4_^+^, NO_2_^–^, NO_3_^–^, SO_4_^2–^, and soluble reactive phosphorus (SRP) (Supplementary Table 2) from each sample were performed as described elsewhere (Martinez-Alonso et al., 2019) by ion chromatography in an 861 Advance Compact IC system (Metrohm AG, Herisau, Switzerland). Ions were identified and quantified with internal and external standards prepared from Certified Standard Solutions (TraceCERT^®^) (Merck, Darmstadt, Germany). Chromatograms were done with the Metrohm IC Net 2.3 SR4 software. Detection limits for constituents that were below detection limits ranged from 0.40 to 2.00 mM.

Concentrations of ions in meltwater (Li, Be, C, F, Ne, Na, Si, P, S, Cl, Ar, K, Ca, Sc, Ti, V, Mn, Fe, Co, Ni, Cu, Zn, Ga, As, Se, Br, Rb, Sr, Y, Nb, Mo, Ru, Ag, Cd, Sb, Xe, Cs, Ba, La, Ce, Pr, Nd, Sm, Eu, Gd, Tb, Dy, Ho, Er, Tm, Yb, Lu, W, Ir, Tl, Pb, Bi, Th, and U) (Supplementary Table 3) were analyzed out by inductively coupled plasma-mass spectrometry (ICP-MS) on a Perkin Elmer ELAN9000 ICP-MS quadrupole spectrometer (Waltham, MA, United States) (Martinez-Alonso et al., 2019). Detection limits for constituents that were below detection limits ranged from 0.040 to 0.300 ppb.

### Extraction, Quantification, and Sequencing of Genomic DNA

The DNA from each 0.22-μm pore filter was extracted by using the DNA Isolation PowerWater kit (MO BIO Laboratory, Inc.). Extraction procedures were identical for all samples. DNA concentration was determined using a NanoDrop 2000p (Martinez-Alonso et al., 2019). Microbial profiles were obtained *via* Illumina MiSeq 16S and 18S rRNA gene amplicon sequencing at the PCM and at the NGS Core Facility (CBMSO, CSIC-UAM). The amplification and sequencing of the V3–V4 regions of the 16S rRNA gene (341F, forward sequence CCTACGGGNGGCWGCAG; 805R, reverse sequence GACTACHVGGGTATCTAATC) were performed to identify Bacteria as previously reported (Herlemann et al., 2011). The classification of bacteria used ClassifyReads against the Greengenes database.

Microeukaryotes were identified by amplification and sequencing of the V4–V5 regions of the 18S rRNA gene (563F, forward sequence GCCAGCAVCYGCGGTAAY; 1132R, reverse sequence CCGTCAATTHCTTYAART) (Hugerth et al., 2014). Quality analyses of reads were performed using FastQ Screen software (v0.12.2.) (Wingett and Andrews, 2018). Paired reads were assembled into one single sequence. This was performed using PANDAseq Assembler (Bartram et al., 2011), which joins the two paired reads correcting sequencing errors in the overlap region, and discards those pairs that do not align between them or that have low quality. This software does also remove the sequence of the primers, discarding the pairs that do not have primer sequences. For the analysis of eukaryotic sequencing data, QIIME2 software was used. The sequences from all of the samples were grouped to define operational taxonomic units (OTUs) using the Silva 138 database. The correspondence of sequences to OTUs was assessed considering a 97% identity threshold using uclust as OTU clustering tool. Both alpha and beta diversity were calculated with QIIME2 software.

The sequences obtained by 16S rRNA and 18S rRNA gene sequencing were deposited in NCBI Sequence Read Archive (SRA) (BioProject PRJNA430179).

### Statistical Analysis

Statistical differences on the number OTUs and diversity indexes (Shannon and Jaccard) were studied by ANOVA test. Data of OTUs were expressed as media ± SEM of three sampling replicates. All of the statistical analyses were studied by ANOVA and Bonferroni’s Multiple Comparison Test using GraphPad Prism version 7.0 (GraphPad Software, La Jolla, California, United States, www.graphpad.com). The data from the sequences were the mean ± SEM from three sampling replicates.

The rarefaction analyses were developed with Analytic Rarefaction 1.3 software^1^ (Tipper, 1979; Supplementary Figure 2).

The effects of the chemical composition of glacial ice, as well as the influence of the age on the microbial community composition, were investigated by a combination of multivariate statistical analysis developed with CANOCO version 5 software (Microcomputer Power, Ithaca) (Jongman et al., 1995; Ter Braak, 1995). Monte Carlo permutation tests with 500 permutations were used to know the significance of regression. Collinear variables were omitted from the analyses.

We firstly performed a DCA for indirect gradient analysis. The gradient length measures the beta diversity in community composition along the ordination axes. In comparison to other available approaches, DCA offers a major advantage: the axes of the DCA space are scaled in standard deviation (SD) units. A distance of 4 SDs indicates a full species turnover; i.e., two sampling sites with an inter-distance of 4 SD or more have no species in common. As the largest value was close to 4, a unimodal method such as CCA was used to search relationships between microbial community structure and environmental parameters, rather than other methods such as PCoA or NMDS (Leps and Smilauer, 2003; Zuur et al., 2007).

## Results and Discussion

### General Characteristics and Chemical Properties of the Glacial Ice

The results of the insoluble suspended matter in meltwater show important changes along the entire profile (ranged from 5 ± 0.6 to 138 ± 5.2 mg), the most relevant one occurring at the MP40 sample (Supplementary Table 1). A slight increase could be detected in the concentration of insoluble particles in samples MP1 and MP10, and between samples MP70 and MP100. No significant differences were found in salinity and pH values among the samples (Supplementary Table 1).

The results from the assays for ions such as NH_4_^+^, NO_2_^–^, NO_3_^–^, SO_4_^2–^, and SRP from each sample are shown in Supplementary Table 2. It is worth highlighting the increase in the concentration of SRP in the samples MP40 and MP50, which coincides with an increase in these samples of insoluble particles (Supplementary Figure 3). These particles that mainly come from the Saharan dust intrusions to Europe constitute an important contribution of nutrients for glacial microorganisms (Cáliz et al., 2018; Pey et al., 2020). Of all the chemical elements that were detected, only those shown in Supplementary Table 3 (C, Na, Si, P, S, Cl, K, Ca, Mn, Fe, Cu, and Zn) presented significant concentrations (≥1 ppb); the remaining were very scarce (<1 ppb) or undetectable. Organic carbon and Zn have been considered potential indicators of current anthropogenic emissions (Moreno et al., 2021). The low concentration of these elements in MPG samples throughout the chronological sequence could indicate their disappearance from glacier surface layers due to its continuous melting associated to last decades. This evidence supports the age model in which the industrial period is not preserved in the ice sequence as a consequence of glacier retreat. Other elements such as Fe, Mn, and Cu can be associated with dust deposition, lithogenic episodes, and mining activities (Callén, 1996; Corella et al., 2018, 2021).

The dating of ice samples, according to Moreno et al. (2021), constitutes a time sequence covering at least the last 2,000 years. Some periods of advance and retreat of ice can be identified. In summary, ice accumulation occurred since the Roman Period (RP) lasting until 1400 years CE. This growth was not homogenous. During the Medieval Climate Anomaly (MCA, 900–1300 years CE), the glacier also suffered a spectacular retreat appreciated by the presence of dark debris layers indicative of the ablation processes. Even if the LIA (1300–1850 years CE) was a period of glacier advance, the ice accumulated during this period cannot be recognized nowadays, since more than 600 years of ice accumulation have been lost due to the warming occurring after 1850 years CE and accelerated in the last few decades.

### Microbial Community Composition

**FIGURE 2:**
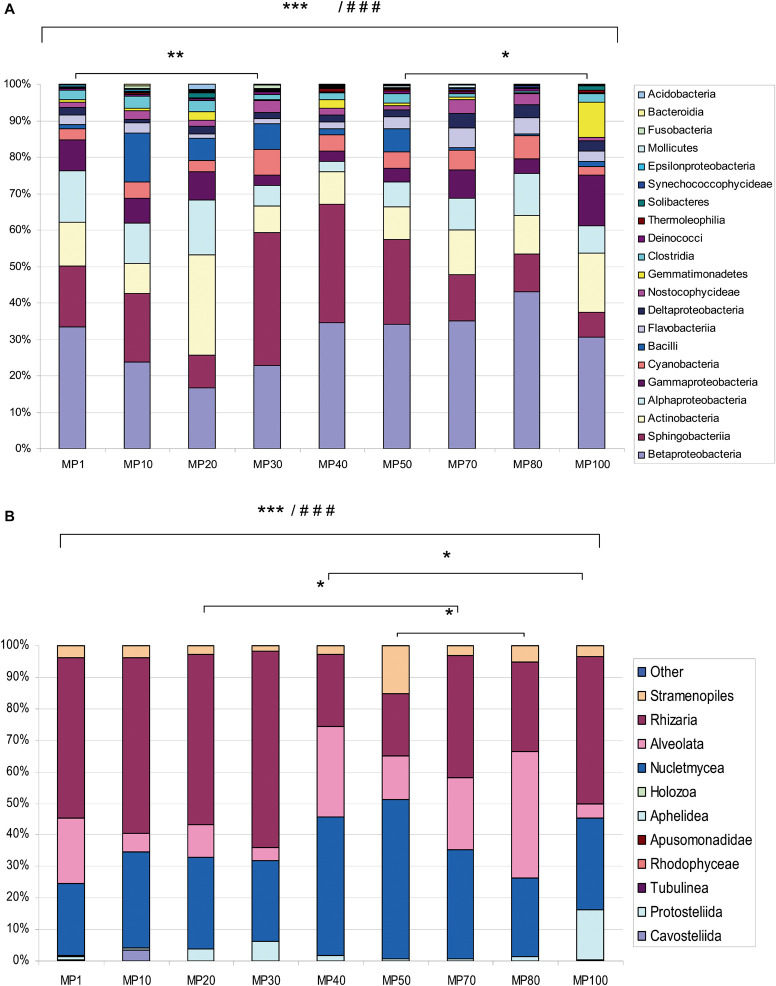
Relative abundances of major classes of bacteria and microeukaryotes. Graphs are based on **(A)** 16S rRNA and **(B)** 18S rRNA gene sequencing data, respectively. Relative abundance is expressed as mean of three sampling replicates per site. The “Other” category is the sum of all classifications with less than 3.50% abundance. Comparison of microbial abundance among samples from MPG analyzed by ANOVA (^###^*p* < 0.0001) and Bonferroni’s Multiple Comparison Test (***p < 0.001; **p < 0.01; *p < 0.05).

The concentration of DNA (ng/μl) extracted from MPG samples was lower in the lowermost samples of the glacial sequence, which correspond with the oldest ones (MP1 15.6 ± 1.5 ng/μl; MP10 13.8 ± 1.1 ng/μl) (Supplementary Table 1 and Supplementary Figure 3). The highest DNA concentrations were found in the MP20 (100.7 ± 3.7 ng/μl) and MP30 (102.8 ± 5.9 ng/μl) samples (Supplementary Table 1 and Supplementary Figure 3). Amplification and sequencing of the V3 and V4 regions of the 16S rRNA gene rendered a total of 1,225,056 reads, which belonged to 1,559 OTUs (Supplementary Table 4) and 56 classes (Figure 2A).

The relative abundance of bacterial classes among samples was analyzed (Supplementary Table 6). ANOVA showed significant differences (^###^*p* < 0.0001). Bonferroni’s Multiple Comparison Test was used to compare abundances between pairs of samples. These values were significant (^∗∗∗^*p* < 0.001), except for MP1 vs. MP30 (^∗∗^*p* < 0.01) and MP50 vs. MP100 (^∗^*p* < 0.05). A significantly higher number of OTUs was observed in MP10 (828 OTUs).

Bacteria mainly corresponded to the classes Betaproteo- bacteria, Sphingobacteriia, Actinobacteria, Alphaproteobacteria, Gammaproteobacteria, Cyanobacteria, and Bacilli. Other classes such as Thermodesulfobacteria, Chrysiogenetes, and Thermoprotei were scarcely represented (Figure 2A). These proportions of bacteria are analogous to those found in glaciers located in similar latitudes such as some Alpine (Franzetti et al., 2017; Azzoni et al., 2018), or Asian glaciers (Chen et al., 2016), but different from those found in the polar glaciers (García-Lopez et al., 2021). Some groups were exclusively identified in the oldest samples as the bacterial phyla Fusobacteria and Calditrichaeota. The relative abundance of some classes varied along the altitudinal transect (Supplementary Figure 4). For example, Betaproteobacteria and Sphingobacteriia show much higher relative abundance in the central zone of the glacier, while Actinobacteria and Alphaproteobacteria were less abundant in that area. In the oldest samples, a greater relative abundance of Fusobacteria and Bacilli was observed. In the most modern samples, the relative abundance was higher in the classes Gammaproteobacteria, Deltaproteobacteria, and Gemmatimonadetes.

Furthermore, the eukaryotic community composition (Figure 2B) was identified by sequencing V4–V5 regions of the 18S rRNA gene. For eukaryotes, 1,263,843 reads were obtained, which corresponded to 1,118 OTUs across 10 phyla spanning 25 classes (Supplementary Table 5). Among eukaryotes, the classes Rhizaria, Nucletmycea, and Alveolata were the most abundant (Figure 2B). The abundance of eukaryotic classes among samples analyzed by ANOVA showed significant differences (^###^*p* < 0.0001) (Supplementary Table 6). The Newman–Keuls Multiple Comparison Test showed that abundances were very different between pairs of samples (^∗∗∗^*p* < 0.001), except for MP100 vs. MP40, MP70 vs. MP20, and MP50 vs. MP80 (^∗^*p* < 0.05). A shifting pattern was also observed in microeukaryote populations (Supplementary Figure 4B). The central area of the glacier contained a greater relative abundance of Stramenopiles, Nucletmycea, and Protostelida. In the older zone, the relative abundance of Rhizaria, Holozoa, Rhodophyceae, and Cavostelida was higher. In the most modern samples, a higher relative abundance of Alveolata, Aphelidea, and Tubulinea was observed.

The diversity of microeukaryotes was similar to that described for other Pyrenean glaciers (Garcia-Descalzo et al., 2013; Cáliz et al., 2018). In these reports, the majority of OTUs belonged to Chlorophyta and Fungi. However, in polar glaciers, the eukaryotic proportions were more diverse and influenced by a range of local factors, so they are not comparable to those of the Pyrenean glaciers (Anesio et al., 2017; García-Lopez et al., 2021).

Test richness, abundance, and diversity were performed by ANOVAs (Supplementary Table 6). Subsequently, Bonferroni post-test was used to test for differences among samples. Statistics indicated that significant differences existed among samples. Considering all possible pairs of comparison of all sampled sites, the mean total beta diversity (Jaccard) showed a gradual turnover along glacial gradient. Multiple comparison test showed that the differences in bacterial populations were very significant for all samples (^∗∗∗^*p* < 0.001). The differences in the eukaryotic populations were significant for all pairs of comparison (Supplementary Table 6).

### Microbial Community Distribution

When the abundance and diversity of the samples along the transect were taken into account, it was observed that there was a gradient with a greater number of OTUs and greater diversity in the oldest samples (Supplementary Table 6).

**FIGURE 3:**
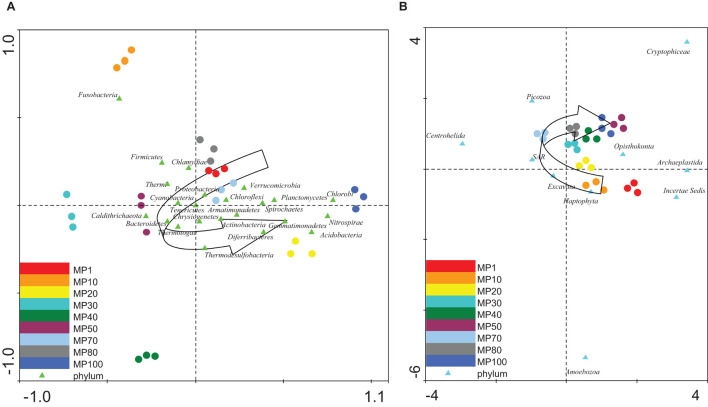
Multivariate statistical analysis. **(A,B)** DCA: The relative abundances of the **(A)** bacterial and **(B)** eukaryotic phyla from glacier samples were compared to find their age gradient. The diagram displays triangles that represent phyla and circles representing samples. Semicircular arrows show how samples follow a trend in the distribution of microorganisms along the age gradient. The axes are scaled in standard deviation units.

In order to determine how the distribution of microbial communities was affected by the chemical properties of the glacial ice, several multivariate statistical analyses of data were carried out. The relative abundance of both bacteria and microeukaryotes at the sampling points was independently analyzed using several Detrended Correspondence Analyses (DCA) (Supplementary Table 7). An age gradient was observed in the distribution of the microorganisms (Figures 3A,B), although some groups of bacteria (i.e., Fusobacteria) (Figure 3A) and eukaryotes (i.e., Amebozoa) (Figure 3B) did not follow this trend.

**FIGURE 4:**
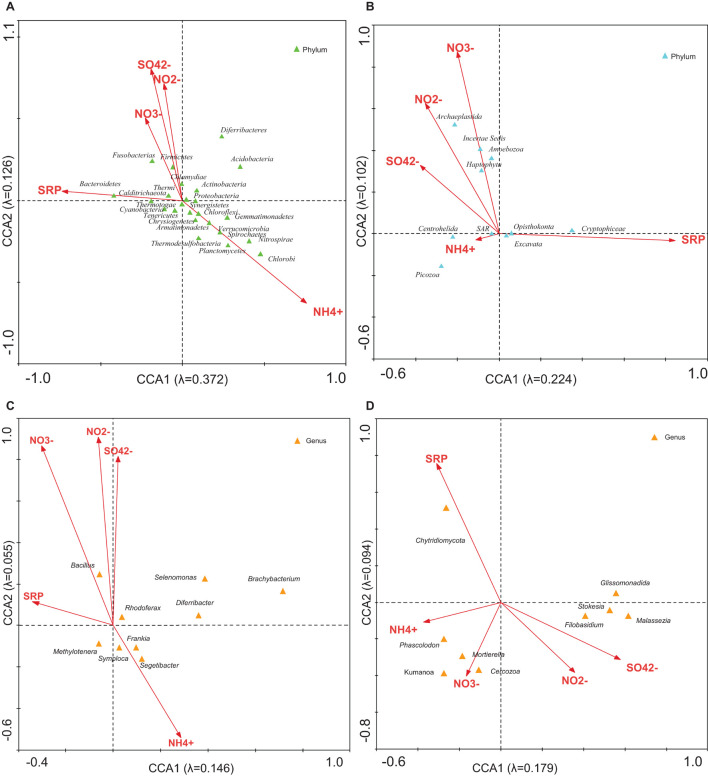
CCA of the microbial groups with regard to the concentration of NH4^+^, NO^2–^, NO^3–^, SO_4_^2–^, and SRP. Analysis of **(A)** bacterial phyla, **(B)** eukaryotic phyla, **(C)** bacterial genera, and **(D)** eukaryotic genera with respect to concentrations. The diagram displays triangles that represent taxonomic groups and arrows that symbolize environmental variables. Correlations between microbial community and ions are represented in axes.

The CCA was used to explore linkages between microbial community structure and environmental parameters. CCA with NH_4_^+^, NO_2_^–^, NO_3_^–^, SO_4_^2–^, and SRP was used to estimate the proportion of the community variability attributable to these ions. It was estimated in several runs. The eigenvalues corresponding to the four ordination axes were used to characterize the results of particular analysis (Supplementary Table 7). CCA diagrams (Figure 4) show the interrelationships between bacterial (Figure 4A) or microeukaryotic phyla (Figure 4B) and these ion concentrations. In analysis no. 2, the CCA produced four axes, which accounted for 52.1% of the total variance in the abundance of bacterial OTUs attributable to these ions. Analysis no. 10 demonstrated that 32.6% of the eukaryotic community variability could be explained by these variables. Some phyla of bacteria such as Firmicutes and Chlamydiae were positively associated with NO_2_^–^, NO_3_^–^, and SO_4_^2–^ (Figure 4A). Some thermophilic bacteria such as Calditrichaeota, Thermi, and Thermotogae were related to SRP (Miroshnichenko et al., 2010; Madigan et al., 2012; Pollo et al., 2015). The most abundant genera of bacteria, *Segetibacter*, *Symploca*, and *Frankia* (Supplementary Table 8) showed an association with NH_4_^+^ levels (Figures 4C,D). Regarding microeukaryotes, Archaeplastida, Amoebozoa, and Haptophyta appeared related with nitrites and nitrates (Figure 4B and Supplementary Table 7). Centrohelida and SAR were associated to ammonium, and other classes such as Cryptophyceae with phosphorus levels. When considering the most abundant genera of eukaryotes (Supplementary Table 8), some of them, including *Cercozoa* and *Chytridiomycota*, were associated to nitrate and phosphate levels, respectively. As mentioned above, these nutrients could come from the Saharan dust intrusions as described in other Pyrenean mountains (Cáliz et al., 2018; Pey et al., 2020).

**FIGURE 5:**
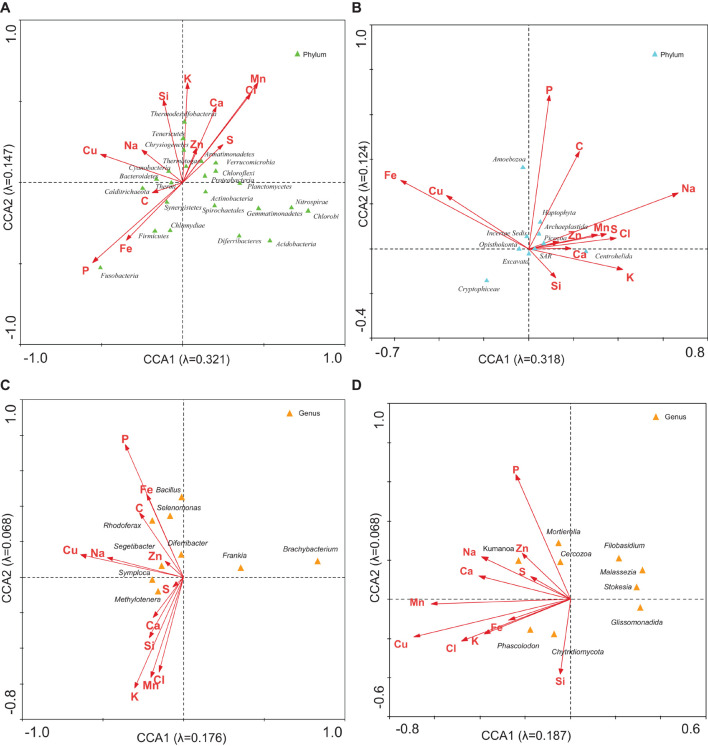
CCA of the microbial groups with respect to the concentration of ions. Analysis of **(A)** bacterial phyla, **(B)** eukaryotic phyla, **(C)** bacterial genera, and **(D)** eukaryotic genera with respect to concentration of C, Na, Si, P, S, Cl, K, Ca, Mn, Fe, Cu, and Zn. Correlations between microbial community and ions are represented in axes.

The CCA with C, Na, Si, P, S, Cl, K, Ca, Mn, Fe, Cu, and Zn (Figure 5; analyses 4, 12) demonstrated that 46.8 and 44.2% of the bacterial and eukaryotic variability could be explained by these variables, respectively. When the age of the samples was added to these variables (analyses 3 and 11), the percentages increased to 66.6 and 54.6%, respectively. This association decreased when only ice age was considered.

It could be observed that the distribution of eukaryotic microorganisms was less related to the concentration of ions or the ice age (analyses 12 and 13) than bacterial distribution was. The absence of a relationship between the abundance of protists and the chemical characteristics of ice had previously been observed in the MPG (Garcia-Descalzo et al., 2013).

### Biochronology of Samples

**FIGURE 6:**
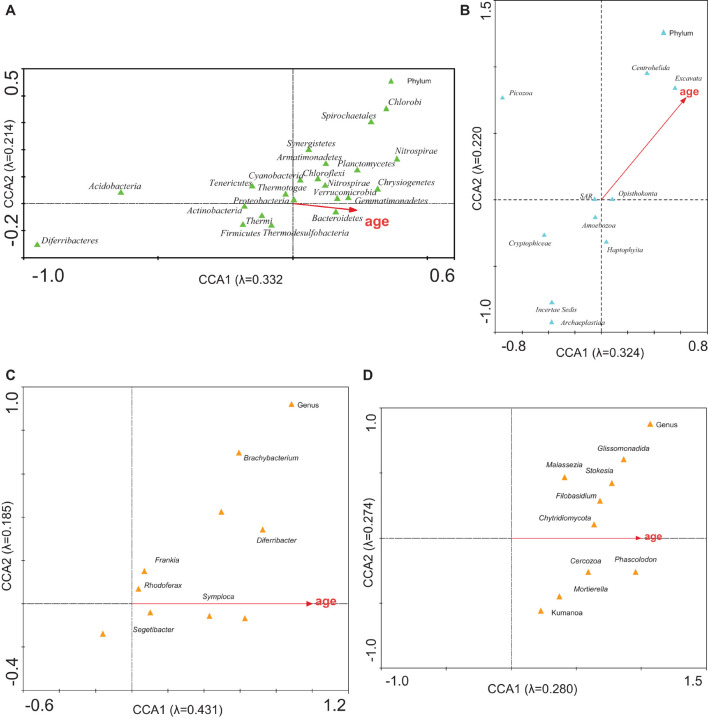
CCA of the microbial groups with respect to age. Analysis of **(A)** bacterial phyla, **(B)** eukaryotic phyla, **(C)** bacterial genera, and **(D)** eukaryotic genera with respect to the ice age. The diagram displays triangles that represent taxonomic groups and arrows that symbolize environmental variables. The axes are scaled in standard deviation units. Correlations between microbial community and age are represented in axes.

When applying the CCA to check the relationship between the microbial community structure and the age of the ice (Figure 6), 54.6 and 54.4% of the bacterial and eukaryotic variability, respectively, were explained (analyses 4, 11, 7, and 14; Supplementary Table 7). Age was mainly associated with some groups of bacteria (Bacteroidetes, Verrucomicrobia, and Gemmatimonadetes) and eukaryotes (Centrohelida and Excavata). These organisms could be markers of the age of the glacier. Bacteria would be better indicators of glacier age than eukaryotes are, because their eigenvalues are higher, than those for eukaryotes (Supplementary Table 7).

Since there is a relationship between the relative abundance of the microorganisms and the dating of the samples, a biochronological graph could be established (Figures 6A,B). Considering that there is a lack of ice from the last 600 years (Moreno et al., 2021), contemporary microorganisms are living on old ice substrates. It is worth asking if these microorganisms have remained quiescent all these years and have now been reactivated, or if they have recently arrived from the environment and the air around the glacier, which indicates that they do not correspond to the age of the ice where they were found.

Microbial populations along the age gradient show different compositions. This may be because they are ancient microorganisms confined to the ice, or that the different ecosystems of the glacier present different states of thaw. It has been shown that microorganisms of different ages react differently to thawing, which eventually leads to distinct bacterial community compositions (Ji et al., 2020).

**FIGURE 7:**
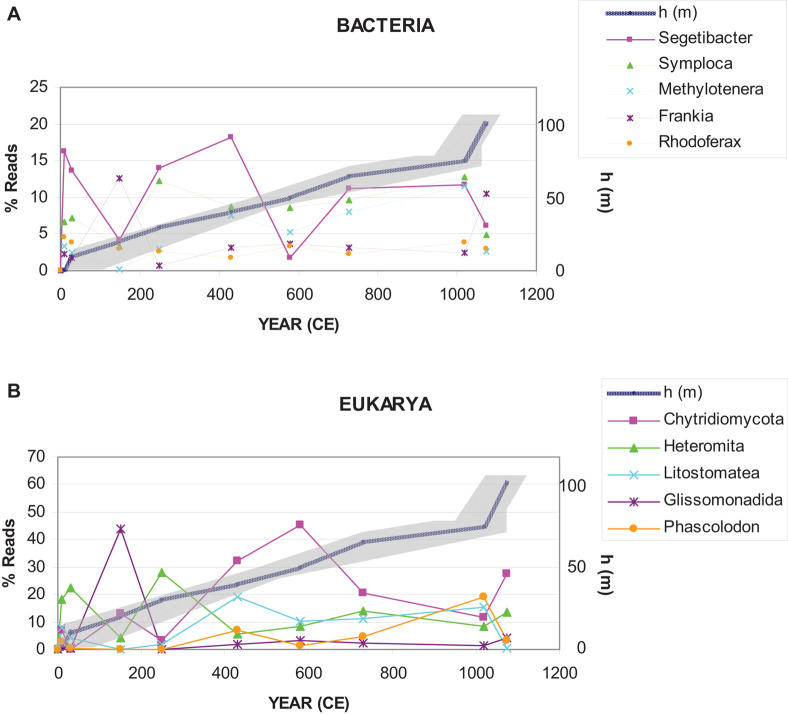
Representation of the most abundant genera of bacteria and microeukaryotes in the ice samples. The depth-age model is based on linear interpolation of ^14^C data calculated in Moreno et al. (2021).

According to previous works (Moreno et al., 2021), comparing the present-day glacier situation with that of previous warm intervals, such as the RP or the MCA, the MPG is nowadays greatly reduced in area and volume. During the RP (0–500 CE), the Iberian Peninsula had a relatively warm temperature (Martín-Puertas et al., 2010), which could explain the observed increase in abundance of the main microorganism representatives, especially *Segetibacter* and U. Chytridiomycota (Figure 7). Later, in the Dark Ages (500–900 CE), the glacier remained active. Most groups maintained a stable abundance, but reduced compared to the previous era. During the MCA, in 900–1300 CE, the glacier retreat and, consequently, the abundance of most groups of microorganisms decreased. This may be due to the increase in temperature and further melting of ice. On the one hand, the water dragged the microorganisms toward the lower part of the glacier; on the other hand, microbial populations came into contact, and consequently, predation and competition increased.

### Biogeochemical Cycles Inferred From Taxonomy

According to the MPG age-depth model, the oldest samples (MP1–MP40) were dated in the Roman Period and Dark Ages. In those times, especially between the 1st and 5th, increased Pb emissions, related to regional mining and smelting activities have been reported (Corella et al., 2018, 2021). Several bacteria such as *Brachybacterium conglomeratum* have been described as copper resistant due to molecular mechanisms (Santo et al., 2010) among which the production of copper-binding peptides stands (DiSpirito et al., 2016). These two bacterial species and others from these two genera were only present in these older ice samples. Other microorganisms adapted to contaminated mine soils (for example, several species of *Selenomonas* and *Bacillus*) were also much more numerous in the ancient samples (Nancharaiah and Lens, 2015; Lusa et al., 2017). Most of the microorganisms found in the oldest samples have an anaerobic metabolism and use sulfate or nitrate as electron acceptors. However, other microorganisms such as *Clostridium*, *Anaerospora*, and *Tepidanaerobacter* (Madigan et al., 2012; Müller et al., 2015) identified in these samples utilize organic electron acceptors.

Among the youngest and contemporary microbiomes, contained in MP70–MP100 samples, some key members of carbon, nitrogen, phosphorus, iron, and sulfur cycles were identified.

Microorganisms that synthesize organic compounds by fixing carbon dioxide (phototrophs and chemolithotrophs) and those that degrade organic matter participate in the carbon cycle. This autotrophic metabolism plays a major role in the global carbon cycle and can help reduce greenhouse gas emissions (Ross et al., 2020). These microorganisms are greatly influenced by the melting of glacial ice layers. Predatory species are numerous in receding glaciers, as they come into contact with their prey that were confined to ice clusters. In these samples, there is a great diversity of ciliates, amoebae, etc. (*Tetrahymena*, *Paramecium*, and *Acanthamoeba*) (Santibáñez et al., 2011; Friman et al., 2016) that feed on both bacterial and eukaryotic primary producers. Glacial ice samples with recent DNA contain a great diversity of photosynthetic organisms such as Cyanobacteria (*Symploca atlantica*). Stramenopiles, phytoflagellates with phototrophic activity, were also abundant, which explains the correlation with N and P (Figures 4A,B; Hodson et al., 2008). Among the chemoorganotrophs, a large number of groups can be cited that metabolize animal and plant remains contained in ice. Several methylotrophs were identified (i.e., *Methylotenera*, *Methylobacterium*, and *Methylobacillus*), which can aerobically catabolize methane and many other carbon compounds. *Methylotenera* has been described as an obligate methylamine utilizer, which uses methylamine as a single source of energy, carbon, and nitrogen (Kalyuzhnaya et al., 2006). Some acetogens, obligate anaerobic bacteria that reduce carbonate to acetate, were also identified (*Corynebacterium acetoacidophilum* and *Acetobacterium malicum*). Among heterotroph microeukaryotes, a high diversity of Rhizaria, Nucletmycea, Alveolata, and Aphelidea was found. Nucletmycea have been extensively described in Arctic and Antarctic soils (Durán et al., 2019; Perini et al., 2019) but are not found so frequently in glacial ice (Butinar et al., 2007). These microorganisms often feed on bacteria or contain other endosymbiotic microorganisms that synthesize vitamins and other growth factors used by the host cell. It has also been described that some Rhizaria can establish photosymbiotic associations (Burki and Keeling, 2014). Aphelidea have been described as intracellular parasitoids of microalgae (Karpov et al., 2014) and diatoms, being sister to true fungi (Letcher et al., 2017).

The main nitrogen cycle representatives in MPG samples were ammonia oxidizers such as *Nitrosococcus* and *Nitrosovibrio* and nitrite oxidizers as *Nitrobacter* and *Nitrospira*.

Some bacteria related to high concentrations of iron and sulfur (Deferribacteres) are chemolithotrophs, able to tap the energy available from the oxidation of inorganic compounds as hydrogen sulfide and ferrous iron. One of the most abundant microorganisms in MPG samples was the Betaproteobacteria *Rhodoferax*, an important Fe^3+^ reducer (Garcia-Lopez et al., 2019). Even so, in recent samples, bacteria adapted to contaminate mine soils are less abundant than in older samples (Supplementary Tables 4, 5).

## Conclusion

This research provides a new high-throughput sequencing data set derived from a receding glacier microbiome. An age gradient was observed in the distribution of the microbial populations. The described microorganisms could be ancient communities in glacier ice or they could come from the upper layers, carried down by the water that percolates through the ice.

Some groups were exclusively identified in the oldest samples as the bacterial phyla Fusobacteria and Calditrichaeota, or the eukaryotic phylum Archaeplastida. Among groups only found in modern samples, the green sulfur bacteria (phylum Chlorobi) stood out, as well as the bacterial phylum Gemmatimonadetes and the eukaryotic class Tubulinea. These proportions are analogous to those found in glaciers located in similar latitudes (i.e., Alpine or Asian glaciers), but different from those found in the polar glaciers.

The oldest samples, from the Roman Empire times, correspond with the beginnings of regional mining activity and contained metal-resistant microorganisms. There is a lack of ice from the last 600 years; contemporary microorganisms are living on medieval ice substrates. With the current climatic conditions, MPG, together with the rest of the Pyrenean glaciers, will disappear. With them, the abundance and diversity of microorganisms they contain will be lost. More scientific attention is needed in order to rescue all the microbiological information that may contribute to understand these unique ecosystems, as they are the basis of food webs and are indicators of environmental health.

## Data Availability Statement

The datasets presented in this study can be found in online repositories. The names of the repository/repositories and accession number(s) can be found in the article/Supplementary Material.

## Author Contributions

CC, AM, and CS conceived and planned the experiments. AM, MB, CS, and ML performed field sampling. EG-L and CC performed lab work and data acquisition. CC, AM, ML, and MB wrote, revised, and edited the manuscript. AM and CC managed the acquisition of funds. All authors have read and agreed to the published version of the manuscript.

## Conflict of Interest

The authors declare that the research was conducted in the absence of any commercial or financial relationships that could be construed as a potential conflict of interest.

## Publisher’s Note

All claims expressed in this article are solely those of the authors and do not necessarily represent those of their affiliated organizations, or those of the publisher, the editors and the reviewers. Any product that may be evaluated in this article, or claim that may be made by its manufacturer, is not guaranteed or endorsed by the publisher.

## Footnotes

1
https://strata.uga.edu/software

## Abbreviations

CCA: Canonical Correspondence Analysis
CE: Common Era
DCA: Detrended Correspondence Analysis
ICP-MS: Inductively coupled plasma-mass spectrometry
LIA: Little Ice Age
MBS: meters below the surface
MCA: Medieval Climate Anomaly
MPG: Monte Perdido glacier
OTU: operational taxonomic units
RP: Roman Period
SRP: Soluble reactive phosphorus.
