# Supplementary material for: Glacial Ice Age Shapes Microbiome Composition in a Receding Southern European Glacier
Source: Front Microbiol. 2021 Nov 11;12:714537. doi: 10.3389/fmicb.2021.714537 (PMC8636055; doi:10.3389/fmicb.2021.714537)

Supplementary Material

Glacial ice age shapes microbiome composition in a receding southern European glacier

*Eva Garcia-Lopez, Ana Moreno, Miguel Bartolomé, Maria Leunda, Carlos Sancho and Cristina Cid**

* Corresponding Author: cidsc@inta.es

This file includes:

I. Supplementary Tables S1-S7

II. Supplementary Figures. Figures S1-S4

I. Supplementary Tables S1-S7

Table S1. Geochemical properties and DNA concentration.

	MP1	MP10	MP20	MP30	MP40	MP50	MP70	MP80	MP100
**pH**	4.9±0.1	5.0±0.5	4.6±0.2	4.8±0.2	4.6±0.9	4.7±0.7	5.0±1.1	4.9±0.8	4.9±0.4
**Salinity (ppt)**	0.31±0.01	0.32±0.02	0.31±0.01	0.31±0.03	0.35±0.04	0.31±0.07	0.30±0.05	0.33±0.01	0.33±0.02
**Insoluble particles (mg)**	44±0.5	26±0.3	5±1.2	5±0.9	138±5.2	3±0.5	5±0.6	25±0.6	17±0.9
**DNA concentration (ng/μL)**	15.6±1.5	13.8±1.1	100.7±3.7	102.8±5.9	89.6±4.7	62.8±2.8	60.6±3.7	87.9±9.4	61.8±8.7

Table S2. Chemical analysis of soluble nutrients in meltwater. Concentrations are expressed as mM (±SEM) of three replicates. [a]BD: below detection.

	MP1	MP10	MP20	MP30	MP40	MP50	MP70	MP80	MP100
NH_4^+	[a]BD	2.01±0.25	4.01±0.30	6.32±1.31	32.66±2.22	61.04±3.22	102.36±5.11	258.32±7.20	481.00±8.02
NO_2^-	70.21±	69. 87±	30.21±	6.88±	5.37±	4.56±	4.22±	3.09±0.26	BD
NO_3^-	101.22±10.22	100.27±9.32	7.33±1.23	6.39±2.36	6.33±1.11	6.01±0.90	5.62±1.99	BD	BD
SO_4^{2-}	373.00±11.21	320.32±12.30	300.98±9.99	201.36±1.25	152.36±3.25	162.35±8.21	174.22±11.09	125.39±5.66	117.00±9.88
SRP[a]	0.47±0.11	0.51±0.06	0.31±0.01	0.86±0.01	1.33±0.23	1.55±0.02	0.42±0.01	BD	BD

[a] SRP: Soluble reactive phosphorus

Table S3. Chemical analysis of ions in meltwater. Concentrations are expressed in ppb (±SEM) of three replicates. [a]BD: below detection.

	MP1	MP10	MP20	MP30	MP40	MP50	MP70	MP80	MP100
C	76.800±11.211	715.441±9.652	63.545±5.222	13.46±2.321	49.497±6.546	58.218±9.548	244.134±10.117	68.438±8.217	22.165±5.210
Na	291.487±19.320	1397.096±54.281	116.482±31.118	171.940±42.111	131.563±10.250	112.306±54.004	206.035±23.872	616.672±29.332	47.213±8.314
Si	14.744±2.350	46.131±7.652	16.630±5.221	9.314±1.258	19.526±4.321	16.206±9.654	22.647±5.654	18.870±2.321	9.374±1.225
P	0.325±0.022	40.705±3.214	[a]BD	6.336±2.333	BD	2.364±0.665	4.339±0.214	0.3637±0.001	0.300±0.003
S	1155.315±25.321	2238.661±54.328	746.387±54.336	658.188±65.357	631.241±41.258	769.398±55.329	756.189±66.217	882.520±7.221	781.192±55.222
Cl	136.600±5.328	375.231±10.287	84.613±8.665	19.465±4.218	96.338±2.225	154.762±11.111	211.301±21.258	391.021±7.654	205.755±5.321
K	40.321±1.225	93.710±11.258	40.766±4.322	53.304±2.258	35.213±1.225	85.433±3.225	75.079±2.228	185.324±7.324	40.649±10.287
Ca	1215.806±14.987	229.985±22.654	111.682±6.327	141.661±9.665	135.402±41.222	315.138±2.258	421.760±25.784	371.227±21.222	270.777±3.698
Mn	1.269±0.254	1.613±0.654	0.236±0.011	0.382±0.099	0.321±0.027	0.838±0.111	1.652±0.999	1.555±0.112	1.296±0.001
Fe	0.763±0.321	6.636±0.001	0.334±0.001	1.091±0.098	0.320±0.121	3.347±0.087	1.131±0.055	0.241±0.011	1.064±0.412
Cu	1.560±0.111	7.653±1.254	0.044±0.001	1.359±0.005	1.484±0.001	3.730±0.001	1.822±0.002	2.360±0.032	0.953±0.011
Zn	221.565±21.368	BD	BD	BD	BD	BD	BD	BD	BD

Table S4. Analysis of bacterial 16S rRNA genes retrieved from ice samples. OTU level aggregate counts of 3 sampling replicates.

File: Table S4.xlsx

Table S5. Analysis of eukaryotic 18S rRNA genes retrieved from ice samples. OTU level aggregate counts of 3 sampling replicates.

File: Table S5.xlsx

Table S6. Number of OTUs and diversity indexes for bacteria and microeukaryotes.

Sample	BACTERIA			EUARYOTES		
	Mean no. of OTUs	Shannon index H´	Jaccard index	Mean no. of OTUs	Shannon index H´	Jaccard index
**MP1**	732	3.221	0.526	424	3.322	0.467
**MP10**	828	3.429	0.452	369	3.340	0.222
**MP20**	635	2.968	0.299	278	2.874	0.125
**MP30**	608	2.568	-0.045	238	2.627	0.10
**MP40**	677	2.654	-0.115	305	2.321	0.111
**MP50**	693	2.843	-0.132	272	1.996	0.091
**MP70**	567	2.683	-0.159	451	2.039	0.011
**MP80**	554	2.743	-0.175	461	2.247	0.005
**MP100**	732	3.221	-0.306	296	1.524	-0.215
**ANOVA P value**	<0.0001***	<0.0001***	<0.0001***	<0.0001***	<0.0001***	<0.0001***
**Multiple comparison** All pairs*** except	-	MP1 vs MP100*	-	MP1 vs MP40**; MP 10 vs MP20*; MP10 vs MP100**; MP20 vs MP100**; MP70 vs MP80**	MP1 vs MP10*; MP30 vs MP40*; MP40 vs MP80*; MP50 vs MP70*; MP50 vs MP80**; MP70 vs MP80**	MP20 vs MP40**; PM30 vs MP40*; MP30 vs MP50*

Statistical differences were studied by ANOVA test on the number of OTUs, Shannon and Jaccard indexes (**, p ≤ 0.001; ***, p ≤ 0.0001). Statistical significance was achieved by Bonferroni's Multiple Comparison Test (*, p< 0.05; **, p ≤ 0.01; **, p ≤ 0.001).

Table S7. Correspondence analysis and Species-environment correlations (λ)

No. of analysis	Type of microorganism	Level	Type of analysis	Environmental variables	λ_1	λ_2	λ_3	λ_4	Figure
1	Bacteria	Phylum	DCA	-	0.430	0.106	0.100	0.000	3A
2			CCA	NH_4^+, NO_2^-, NO_3, SRP, SO_4^{2-}	0.372	0.126	0.014	0.009	4A
3				Age, C, Na, Si, P, S, Cl, K, Ca, Mn, Fe, Cu, Zn	0.485	0.142	0.023	0.016	-
4				C, Na, Si, P, S, Cl, K, Ca, Mn, Fe, Cu, Zn	0.321	0.147	0.010	0.000	5A
5				Age	0.332	0.214	0.000	0.000	6A
6		Genus		NH_4^+, NO_2^-, NO_3, SRP, SO_4^{2-}	0.146	0.055	0.014	0.006	4C
7				C, Na, Si, P, S, Cl, K, Ca, Mn, Fe, Cu, Zn	0.176	0.068	0.019	0.011	5C
8				Age	0.431	0.185	0.044	0.016	6B
9	Eukarya	Phylum	DCA	-	0.446	0.113	0.101	0.000	3B
10			CCA	NH_4^+, NO_2^-, NO_3, SRP, SO_4^{2-}	0.224	0.102	0.000	0.000	4B
11				Age, C, Na, Si, P, S, Cl, K, Ca, Mn, Fe, Cu, Zn	0.343	0.102	0.066	0.035	-
12				C, Na, Si, P, S, Cl, K, Ca, Mn, Fe, Cu, Zn	0.318	0.124	0.000	0.000	5B
13				Age	0.324	0.220	0.000	0.000	6C
14		Genus		NH_4^+, NO_2^-, NO_3, SRP, SO_4^{2-}	0.179	0.094	0.030	0.003	4D
15				C, Na, Si, P, S, Cl, K, Ca, Mn, Fe, Cu, Zn	0.187	0.097	0.024	0.005	5D
14				Age	0.280	0.274	0.150	0.078	6D

Table S8. Number of sequences belonging to the most abundant genus in each sampling point

Bacteria									
	MP1	MP10	MP20	MP30	MP40	MP50	MP70	MP80	MP100
Genus[a]	*Segetibacter*	*Segetibacter*	*Frankia*	*Segetibacter*	*Segetibacter*	*Segetibacter*	*Segetibacter*	*Symploca*	*Frankia*
No. sequences[b]	6680	9746	6368	6396	10865	8065	6598	6130	5516

Eukarya									
	MP1	MP10	MP20	MP30	MP40	MP50	MP70	MP80	MP100
Genus[a]	U.Cercozoa	U.Cercozoa	*Glissomonadida*	U.Cercozoa	U.Chytridiomycota	U.Chytridiomycota	U.Chytridiomycota	*Phascolodon*	U.Chytridiomycota
No. sequences[b]	20890	11060	76908	19004	69106	80437	36098	33857	31509

[a]Most abundant genus in each setting

[b]Number of total sequences from the most abundant genus in each sampling point

II. Supplementary Figures.

Figure S1. Summary of the overall experimental strategy

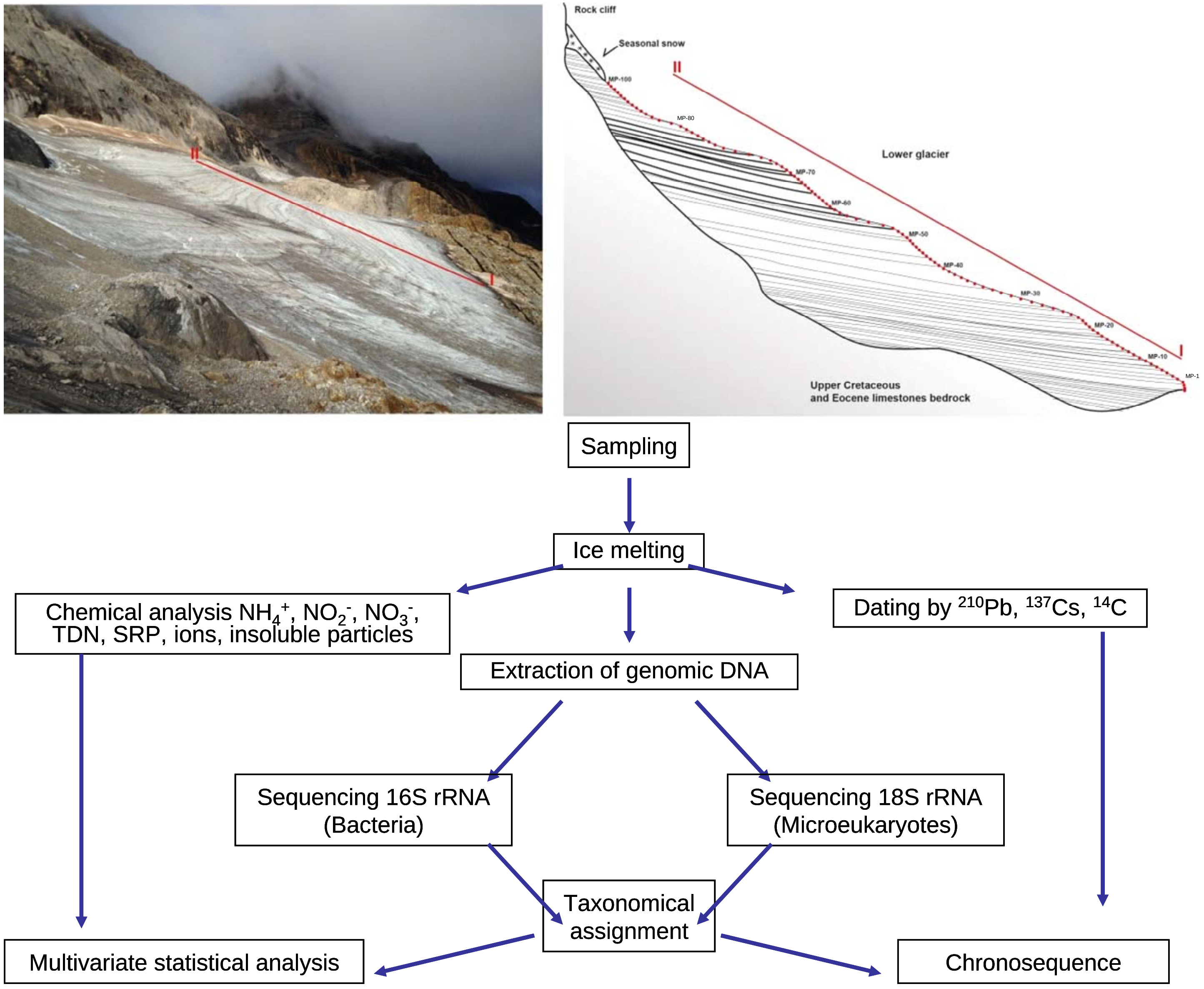

Figure S2.

Rarefaction curves determined for 16S rRNA and 18S rRNA gene clones. Rarefaction curves for bacteria and microeukaryotes indicating the observed OTUs at a genetic distance of 3%.

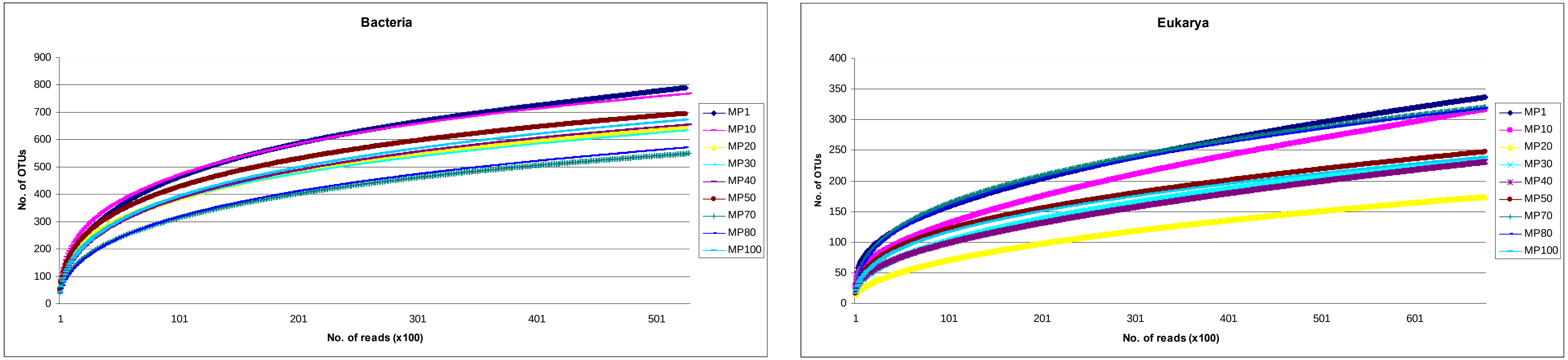

Figure S3. Sample characterization. Content of insoluble particles, DNA and secuences in MPG samples along a 100 m altitudinal transect.

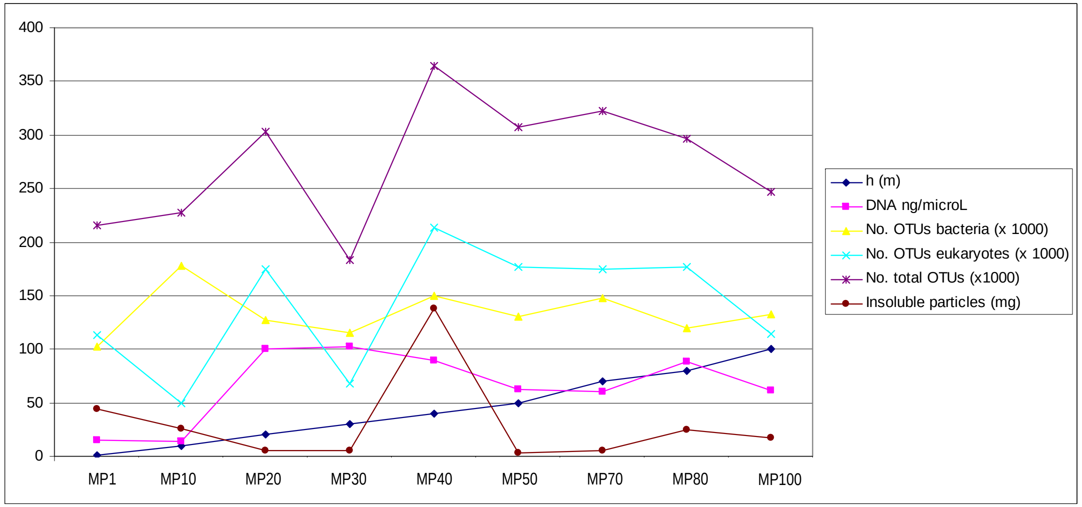

Figure S4. - Examples of the shift of main taxa of bacteria and microeukaryotes in the glacier samples. Graphic representation of the number of 16S rRNA and 18S rRNA sequences (in log) and their corresponding trend lines.

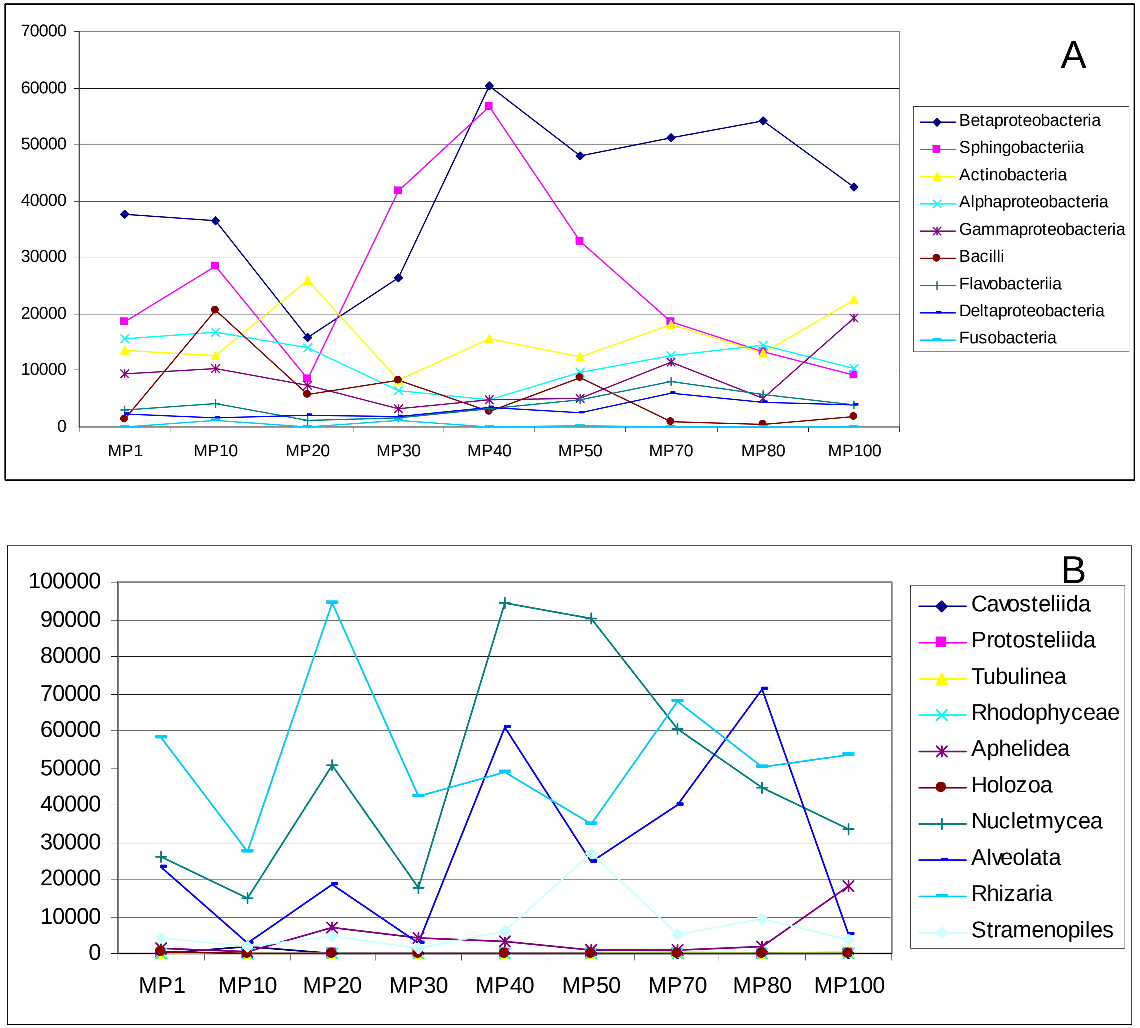

Supplement: Supplementary file 1 [file Data_Sheet_1.pdf]
